# Research to Move Toward Evidence-Based Recommendations for Lead Service Line Disclosure Policies in Home Buying and Home Renting Scenarios

**DOI:** 10.3390/ijerph16060963

**Published:** 2019-03-18

**Authors:** Hang Lu, Rainer Romero-Canyas, Sofia Hiltner, Tom Neltner, Lindsay McCormick, Jeff Niederdeppe

**Affiliations:** 1 Annenberg Public Policy Center, University of Pennsylvania, Philadelphia, PA 19104, USA; hang.lu@appc.upenn.edu; 2 Office of Chief Scientist, Environmental Defense Fund, New York, NY 10010, USA; rromero@edf.org (R.R.-C.); shiltner@edf.org (S.H.); 3 Program in Environmental Health, Environmental Defense Fund, Washington, DC 20009, USA; tneltner@edf.org (T.N.); lmccormick@edf.org (L.M.); 4 Department of Communication, Cornell University, Ithaca, NY 14853, USA

**Keywords:** lead service lines, scenario-based experiment, property disclosure style, home inspector, risk perception

## Abstract

Lead service lines (LSLs)—lead pipes connecting the water main under the street to a building’s plumbing—contribute an estimated 50% to 75% of lead in tap water when they are present. Although Congress banned lead in plumbing materials in 1986, over 6 million LSLs remain in homes across the United States today. This paper summarizes three different home buying or renting scenario-based experimental studies used to evaluate disclosure styles, to assess if these influenced respondents’ perceived risk of the LSL in a home, and their willingness to act. In renting scenarios, having landlords disclose the presence of an LSL, but also provide water test results showing lead levels below the EPA’s lead action level resulted in lower levels of perceived risk, and of willingness to act. In seller-disclosure home buying scenarios, levels of perceived risk and willingness to act were consistently high, and three different disclosure styles did not differentially influence those outcomes. In home inspector-disclosure home buying scenarios, levels of perceived risk and willingness to act were high, but having explicit recommendations to replace LSLs and/or information about risk did not further influence those outcomes. In some cases, including the specific recommendations backfired. Implications for policy and regulation are discussed.

## 1. Introduction

There is scientific consensus that there is no safe level of lead in children’s blood [1]. The tragedy of Flint, Michigan in 2014 to 2017, in which many children were exposed to substantial and toxic levels of lead via changes to the municipal water source, brought renewed attention to the health threats posed to children by lead in general, and by lead in drinking water in particular [2]. Widespread attention to this crisis prompted the United States (U.S.) Federal government and many states to reassess their existing strategies to protect children from lead, and to search for opportunities to reduce exposure from all sources [3].

Homes are a major source of lead exposure in the US. Lead is often found in the home in the form of lead-based paints or lead service lines (LSLs)—lead pipes that connect the main water line under the street to a building’s plumbing [4]. When present, LSLs contribute the greatest percentage of lead—an estimated 50% to 75%—to the drinking water in a home [5]. Although both sources are significant, the relative contribution of lead in paint or water to the overall exposure varies by the child’s age, but contribution by paint is generally greater [4]. Although Congress banned the use of lead in paint in 1978, and in plumbing materials in 1986, an estimated 37 million U.S. homes have lead-based paint [6], and an estimated 6 to 10 million homes have LSLs today [7]. Most homes with LSLs would likely also have lead-based paint, based on a home’s age. While U.S. policy has focused far more on exposure to lead-based paint than on LSLs, the crises with lead in water in Washington, DC in the 2000s garnered significant attention toward LSLs, and the Flint tragedy crystallized the demand for action [8,9].

### 1.1. Lead-Based Paint Disclosure Policy in the U.S.

Since 1996, Federal law has required that property owners disclose the presence of lead-based paint and related hazards to potential home buyers and renters for residences that are built before 1978, the year of when the sale and use of new lead-based paint was prohibited [6]. This policy assumes that people seeking to rent or to buy a home can access the information that they need, in order to make an informed decision about protecting their families from lead-based paint. As a result, they could demand that the property owner either remediate the hazards, or adjust the price to enable the buyer or renter to take action to reduce lead risks on their own [10]. However, the seller is not required to determine whether lead-based paint or related hazards are present in the home, if that information is unknown. If the seller does not have that information, the buyer can choose to pay for a home inspection or risk assessment, or they can look for another home. In promulgating this policy, the federal government reasoned that market demand would result in property owners proactively evaluating homes and eliminating lead hazards [10]. Federal agencies saw the rule as an essential means to correct a “market imperfection” caused by a lack of available information to prospective home buyers and renters [10].

This policy has not eradicated the problem. As noted above, around 37 million homes have lead-based paint [11]. While the disclosure requirement has raised awareness about lead-based paint, lead poisoning prevention advocates have singled it out as being ineffective at correcting the market imperfection, because there is no mandate to test for lead in homes, and as a result, very few homes have been inspected for the presence of lead-based paint [12]. The details of the policy in place create a paradox. The only persons who are allowed by law to inspect for lead-based paint for compensation are people who have completed a 5-day training course, passed a test, met experience or education requirements, and have become certified lead-based paint inspectors by the EPA or the state. Few home inspectors will make such an investment without being confident that there are sufficient homebuyers who are willing to pay the $600 to $1000 fee for the service. At the same time, those homebuyers who may be willing to pay, often cannot find a certified person who can do the work in a timely manner without charging a premium. Furthermore, most homebuyers do not want to risk delaying a closing, especially if they are not certain what they would do if it called for major remediation that might run into tens of thousands of dollars. As a result, a lead-based paint inspection is usually only conducted as part of an environmental investigation conducted after a child with an elevated blood lead level is found [13]. 

### 1.2. Lead Service Line Disclosure Policy in the U.S.

These federal disclosure requirements apply only to lead-based paint, and not to the lead in service lines or household plumbing. In a 2017 review of state policies, the Environmental Defense Fund (EDF) found that only seven states require sellers to disclose the presence of lead in plumbing [14]. More than 20 other states only require disclosure if the seller considers the lead pipe to be in an “unsafe condition” or an “environmental hazard or defect” [14]. In addition, three states (Kentucky, Minnesota, and New York) require the disclosure of drinking water test results, if available [14]. For renters, there are lead pipe disclosure requirements for landlords in the cities of Cincinnati, OH, Philadelphia, PA, and Washington, DC, but to our knowledge, this is not the case for any other municipalities or states [15].

Despite the challenges observed with lead-based paint disclosure, the National Drinking Water Advisory Council (NDWAC) for the EPA recognized the essential role of disclosure during property sales when the agency asked for its recommendations to revise the agency’s rule, which is designed to limit lead in drinking water (Lead in Copper Rule). As part of a comprehensive program to reduce lead exposure, NDWAC called for a long-term effort to eliminate LSLs, and for states to “pass legislation requiring inspection, disclosure and/or replacement of LSLs on sale of property, and when lines have been disturbed as part of a renovation” [16,17]. It also recommended that the EPA require drinking water utilities to tell customers—typically the property owner—whether they have an LSL. If passed, such a requirement would address a key limitation of the lead-based paint disclosure—the lack of a mechanism, or an incentive for a home seller or a landlord to find out whether they have lead hazards in the first place.

Recognizing this and other shortcomings of lead-based paint disclosure, in 2016, the EPA called on states to make publicly available the locations of LSLs [18]. It is also considering making it a requirement for drinking water utilities to develop inventories of LSLs, to notify individual customers if they have a lead pipe, and to make the addresses public. Several communities now make interactive maps of LSLs that are available online, and in a separate study, we explored the effectiveness of various options to make address-specific information on LSLs publicly available [15]. In contrast to lead-based paint, a home inspector should be able to find out if an LSL is present fairly easily, by contacting the utility, and by inspecting the service line as it enters the home. 

### 1.3. Key Questions for LSL Disclosures in Home Buying/Renting Scenarios

Given these developments, it seems likely that an increasing number of property owners will have access to information about the presence or absence of an LSL on their properties. In response, property owners may test water for lead, to assess the risk, and to compare it to the EPA’s Lead Action Level (LAL) of 15 parts per billion. This comparison is misleading, because the LAL is not a health-based standard; rather, it is designed to evaluate the water utility’s ability to treat water to reduce corrosion on a system-wide basis [19]. Further, the complex and unpredictable nature of lead in water testing may miss lead peaks, providing negative results when there is in fact a risk of lead exposure.

The disclosure of LSLs to property owners, in and of themselves, is not sufficient to mitigate the threat of lead exposure; it must be followed by the replacement of the entire service line. Replacing LSLs is challenging, however, because the ownership of the service line is often shared between the utility (the “public side” or “city side”) and the property owner (“private side”). As a result, in many communities, residents or property owners are responsible for funding all or part of an LSL replacement, which can cost thousands of dollars.

The sale or rental of a home, particularly if disclosures are mandated, provides a unique opportunity for the owner and/or buyer/renter to replace the LSL as a condition of the rental or sale. As noted above, the LSL disclosures could come from one of several sources, depending on the home buying or renting scenario: (a) the landlord in the rental of a home, (b) the seller of a home, and/or (c) a home inspector in a home sale. We identified no evidence-based guidance on communication strategies for disclosures about the presence of an LSL that maximizes the likelihood of replacing an LSL on private property.

### 1.4. Research Questions and Hypotheses

**Figure 1:**
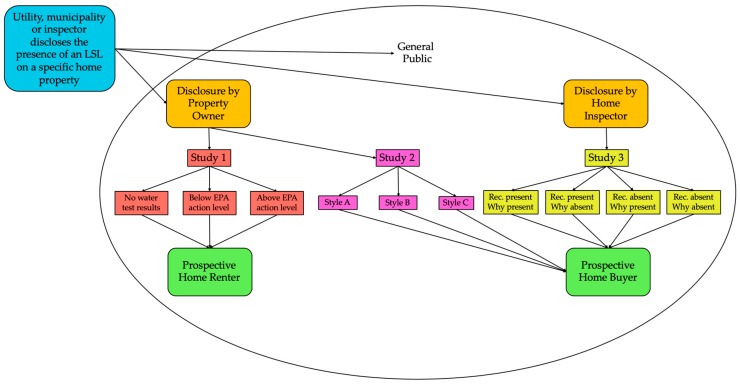
Summary of home renting and buying scenarios. LSL: Lead service lines. Rec.: Recommendation.

In light of these questions about which disclosure practices most effectively motivate action by the property owner or buyer/renter to replace an LSL, we sought to develop evidence-based recommendations for LSL disclosure policies in key scenarios, by evaluating responses among potential home buyers and home renters to being told that a home has an LSL by the property owner or a home inspector. We tested the effects of different disclosure language styles and content, for each of three scenarios (Figure 1) on a variety of outcomes that included the willingness to adopt risk mitigation behaviors, risk perceptions, self-efficacy (the perception that the individual has the ability and resources to avert a threat), response efficacy (the perception that various actions will be successful in reducing the threat), perceived affordability, and seeking additional information about LSLs. The disclosure styles that we tested were based on language and formatting that was actually in use, or that were being considered for use. In the case of the home inspector disclosure, we modeled the disclosure on typical inspector reports.

For Study 1 (landlord disclosure to home renter), we compared responses to (1) a simple disclosure of an LSL, (2) a disclosure accompanied by a water test that showed results below the EPA’s lead action level, and (3) a disclosure with a water test that showed results above the EPA’s lead action level. Following previous research examining how people respond to additional alarming or reassuring information about a risk topic [20], we conceptualized the information about testing results above the action level as being alarming, and the information about the test results below the action level as being reassuring. Based on prior research [20], we hypothesized that the reassuring disclosure with a “Below EPA level” test result would lead to a lower willingness to adopt effective risk mitigation behaviors (demanding that the landlord replace the LSL, or choosing another rental property) than the other two conditions (alarming or simple disclosure). The renter was not provided with any information about how the water was sampled, the significance of the EPA lead action level, or the limitations of testing water for lead.

For Study 2 (seller disclosure to buyer), we compared responses to three styles of lead disclosure forms used in states across the country: one disclosing that “lead plumbing” is present, one disclosing that “lead hazards” are present and specifying an LSL connected the water main under the street to the home, and one disclosing the presence of an “environmental hazard” and specifying an LSL connected the water main under the street to the home. All three forms disclosed, in some format, the presence of an LSL. We did not provide an option for “no LSL” or “LSL unknown” because these options are not included as part of typical disclosures, and in this study we chose to focus on the effects of various disclosure styles, given the known presence of an LSL by a landlord, home seller, or home inspector. Framing research has shown that small variations in the presentation of an issue can influence audience knowledge and perceptions [21]. We did not have any *a priori* hypothesis about which disclosure style would have the largest effect on willingness to adopt effective risk mitigation behaviors, but we sought to identify which of the existing state approaches to LSL disclosures would be the best candidate wording for wider adoption.

For Study 3 (home inspector disclosure to buyer), we compared responses to four versions of a home inspection report that included information about the presence of an LSL on the property. Each version disclosed the presence of an LSL, and some conditions included additional information about “why to replace an LSL”, as well as explicit recommendations about strategies to ensure the removal of the LSL before moving into the home. We based our options in Study 3 on a recent meta-analysis [22] investigating the effectiveness of fear-appeals, which showed that messages were more effective at positively influencing behavioral intentions when they depicted the severity of the risk as being higher (vs. low) and when efficacy statements were present (vs. absent). We conceptualized the “why” information (described below) as being high-severity information about lead, and the explicit recommendations as statements that might influence people’s perceived efficacy, because tangible actions to remove LSLs were provided. In other words, high-severity risk messages, accompanied by suggested actions to counter the risk should elicit more willingness to act. Hence, we hypothesized that the conditions with “why” information would produce a greater willingness to replace the LSL prior to moving in, than the conditions without it, and that the explicit recommendation would increase the willingness to replace the LSL, relative to the conditions without such a recommendation.

Within each study, we also explored which of the possible responses to learning about the presence of LSLs were preferred by respondents, either in the situations where they were buyers or renters of new homes. While we had no specific hypotheses, we expected that in general, respondents would seek to minimize their exposure to lead, and to reduce the financial burden of doing so. We also explored the question of whether any form of disclosure would lead to them passing up on the opportunity of purchasing or renting a home. 

## 2. Materials and Methods

### 2.1. Participants

We recruited participants for all three experiments in one wave, through Amazon’s Mechanical Turk (M-Turk), an online participant recruitment tool. We described our study as “Answer a survey about an important topic” without any mention of lead, when we posted the survey link on M-Turk. We required participants to be adults living in the United States who had previously gone through the process of either buying or renting a home. We used this inclusion criterion to ensure that participants would be familiar with the home-buying or rental processes, since the questionnaires included questions focusing on negotiations in various stages of the process of buying or renting. 

We collected data on 12 April and 13 April 2018. We paid participants who met the inclusion criterion 1 U.S. dollar for completing the experiment. We paid five cents to those who did not meet the inclusion criterion, but who answered the screening questions; we excluded them from the study. The final sample included 2205 participants, with 667 in the landlord/renter study, 667 in the seller/buyer study, and 871 in the inspector/buyer study.

The total sample was 56.2% female, with an average age of 39.4 years. The median level of education was that of a bachelor’s degree, and the median level of income corresponded to between $50,000 and $74,999 in annual household income. The majority (84.2%) of respondents identified themselves as white, with no other race or ethnicity selected. In terms of political ideology, over half (51.1%) of the participants identified as liberals, 19.4% as moderates, and 29.5% as conservatives. 40.8% of participants identified themselves as being Democrats, 23.1% as Republicans, and 29.4% as Independents. Over half of the sample (51.9%) had children, but only 33.1% reported living with a child aged 12 or under. Over a quarter of the sample (26.5%) had heard about LSLs before taking the survey. Table 1 depicts the demographics within each study sample.

### 2.2. Procedure and Stimuli

After answering the screening questions, we assigned all respondents into one of three scenarios. In all three studies, we first asked participants to imagine that they have been looking to either rent a home (for those in the landlord/renter study), or to buy a home (for those in the seller/buyer or inspector/buyer studies), and then to write a few sentences about what their perfect home may look like. This introduction was designed to immerse them in a hypothetical scenario (see Supplementary Files S1–S3 for the full text of all of the LSL disclosure conditions, and the questionnaire).

Respondents who had not purchased a home before, but who had experience in renting a home proceeded to Scenario 1 (“Study 1”, the “landlord/renter” study). We asked Study 1 participants to imagine that they had already applied to rent their perfect home, and the landlord has accepted their application. We then randomly assigned Study 1 participants, to see one of three versions of disclosure information about the presence of an LSL from the landlord. The “No Water Test Results” version told participants, “The landlord has disclosed that there is a lead service line on this property”. The “Below EPA Level” version featured the same LSL disclosure, but it also told participants that the landlord had provided lead water test results, indicating that the level was lower than the EPA’s lead action level. The “Above EPA Level” version also featured the same LSL disclosure, but told participants that the landlord had provided lead water test results, indicating that the level was higher than the EPA’s lead action level. S1 depicts the full text of these disclosures.

Respondents with previous experience purchasing a home were randomly assigned to respond to one of two home-buying scenarios. Scenario 2 (“Study 2”, the “seller/buyer” study) focused on a seller disclosing an LSL, while Scenario 3 (“Study 3”, the “inspector/buyer” study) focused on a home inspector disclosing the presence of an LSL. 

We asked Study 2 participants to imagine that they have already filed the paperwork and made an offer to buy their perfect home, and that the seller had accepted their offer. We then showed participants a brief section from the disclosure statement, where the presence of LSLs would be disclosed. There were three versions of this section, corresponding to the three styles of housing disclosure forms used in states across the country. We randomly assigned each participant to see only one of the three versions—Style A, Style B, or Style C. Style A included the question, “Is lead plumbing present?” Style B included the question, “Are there any lead hazards? (e.g., lead paint, lead pipes, lead in soil.)” Style C included the question, “Are you aware of any substances, materials, or products that may be an environmental hazard, such as asbestos, formaldehyde, radon gas, lead-based paint, fuel, or chemical storage tanks, contaminated soil, water, or by-products from the production of methamphetamines on the subject property?” In all three versions, the “yes” box in response to the question was checked, and the issue was further described as being “lead service line connecting main under street to home”. We included these three styles of disclosure, as they are representative of those that are currently in use.

We also asked Study 3 participants to imagine that they have already filed the paperwork, and that they had made an offer to buy their perfect home, and that the seller had accepted their offer. In these scenarios, the seller did not disclose anything upsetting about the home in the mandatory disclosure forms, but the buyer had arranged for a home inspection. We then showed participants a summary of this home’s inspection report, which identified two issues with the home—energy inefficient windows on one floor, and the presence of an LSL on the property. The information on energy inefficient windows was the same for all participants in this experiment, and it was designed to make the inspection report seem more credible in identifying more than one issue with the home (with the replacement costs being estimated to be similar to that of replacing the LSL). 

We randomly assigned Study 3 participants to see only one of four versions of the LSL portion of the inspection report. All Study 3 versions of the inspection report included a short paragraph, with additional information about window replacement (along with a cost-estimate of between $1000 to $5000 per floor). All of the versions also included a paragraph defining LSLs, a brief description of the cost of replacement ($1000 to $5000, consistent with the typical cost in the U.S.), and an explicit statement that the removal of an LSL substantially reduces the risk of lead exposure, and should increase the market value of the home. In addition, the “Recommendation Present, Why Present” version made explicit recommendations about specific actions that could be taken to replace LSLs (adding the cost of replacement to the mortgage, deducting the cost of replacement from the sale price, and/or demanding that the seller replace prior to closing on the home), and included bulleted information about why it was important to replace LSLs. The “why” information stated there is no safe level of exposure to lead, and described the specific health effects of lead exposure for children (impaired brain development, learning and behavioral problems, and lower IQ), adults (cardiovascular effects, increased blood pressure, and incidence of hypertension) and pets (problems related to the gastrointestinal tract and central nervous system). The “Recommendation Present, Why Absent” version provided only the recommendations (without the “why” information). The “Recommendation Absent, Why Present” version provided only the “why” information (without any recommendation). The “Recommendation Absent, Why Absent” version provided neither the recommendations, nor the “why” information. 

Immediately after these disclosures, we provided all participants in Studies 1, 2 and 3 with a link to an online PDF version of the 20-page pamphlet, “Protect Your Family from Lead in Your Home [23]”. The pamphlet, created by the U.S. Environmental Protection Agency, Consumer Product Safety Commission, and Department of Housing and Urban Development, is required to be provided to the buyer or renter of a property built before 1978 before they sign a contract. Participants were told that they could choose to look at this pamphlet, which includes information about the risks of lead in drinking water on page 13, or proceed to the next page. We included the link to the pamphlet to mimic a real-world scenario, where providing a hard copy of this pamphlet to home buyers or renters is required by federal law. We recorded whether or not participants clicked on the link. Finally, participants were instructed to think about the home buying or renting scenario, and the information that they have been provided when answering survey questions on the following pages.

### 2.3. Measures

#### 2.3.1. Willingness to Adopt Risk Mitigation Behaviors

We asked our participants to rate their willingness to adopt a series of behaviors regarding the LSL in the new home. We included five items in the landlord/renter study (Study 1) and seven items in the seller/buyer (Study 2) and inspector/buyer (Study 3) studies. The response scale ranged from 1 to 6, and it was anchored at every point (e.g., 1 = extremely unlikely, 6 = extremely likely). Table 2 includes more detailed descriptions of each of the measures included in this set of items. We treated each of these items individually, because some of these behaviors are likely to be effective at reducing the risk of exposure to lead in the home, while others are not likely to be effective, particularly those that ignore the problem entirely. For instance, in the landlord/renter study, RB1 (Renter-Behavior 1) and RB2 (which describe different strategies for LSL removal) are the most desirable behaviors from the perspective of reducing risk of lead exposure, because they ensure that the LSL will be removed entirely. RB5 (look for a home elsewhere) reduces the risk to the current potential renter, but leaves the LSL in place, relying on the landlord to replace the LSL to increase the home’s marketability. This should create a market incentive to replace the LSL. RB3 (lead water filters) could be effective at reducing lead exposure, but they must be replaced regularly, they reduce the speed of water flow to levels that some people find undesirable, and they ultimately leave the LSL in place for future renters. RB4 (leave it alone) is the least desirable response, because it would do nothing to reduce lead exposure, and it would not create a market-based incentive for replacement. 

In the seller/buyer and inspector/buyer experiments, BB1 (Buyer-Behavior 1), BB2, BB3, and BB4 (which again describe different strategies for LSL removal) are the most desirable behaviors for reducing lead exposure in the home, in perpetuity. The “Recommendations present” conditions in the home inspector disclosure study made explicit recommendations for BB1, BB2, or BB3. BB5 (lead water filter), and BB7 (look elsewhere) are less desirable for the same reasons described above. BB6 (leave it alone) is undesirable, since it leaves the exposure unchanged, and it does not incentivize the seller to replace the LSL. 

#### 2.3.2. Risk Perceptions

All three studies included five items to assess the perceived severity (e.g., “Exposure to lead has serious negative consequences”) and susceptibility (e.g., “Lead service lines in my home would expose me to lead in my drinking water”) of exposure to lead [24]. The response scale of this measure ranged from 1 to 6, and it was anchored at every point (e.g., 1 = Strongly disagree, 6 = Strongly agree). We averaged these five items to create a risk perception scale. The means were similar for all three studies. (Study 1: *α* = 0.81, *M* = 5.04, *SD* = 0.73; Study 2: *α* = 0.85, *M* = 5.09, *SD* = 0.79; Study 3: *α* = 0.86, *M* = 5.19, *SD* = 0.81).

#### 2.3.3. Response Efficacy

We adopted two items, including “Having lead pipes replaced with lead-free ones would significantly reduce the exposure to lead for people in the new home”, and “One way to significantly reduce exposure to lead in drinking water is to replace the lead service line with a lead-free line”, to assess the response efficacy [24]. The response scale of these measures ranged from 1 to 6, and it was anchored at every point (e.g., 1 = Strongly disagree, 6 = Strongly agree). We averaged the two items to create a response-efficacy scale. As with risk perceptions, the means were similar for all three studies. (Study 1: *r*_SB_ = 0.75, *M* = 5.16 *SD* = 0.93; Study 2: *r*_SB_ = 0.84, *M* = 5.24, *SD* = 0.91; Study 3: *r*_SB_ = 0.83, *M* = 5.38, *SD* = 0.86).

#### 2.3.4. Self-Efficacy

We used two items, including “I would be able to have lead pipes replaced with lead-free ones in the new home”, and “I don’t think that I could have the lead pipes replaced with lead-free ones in the new home”, (reverse coded), to measure self-efficacy [24]. The response scale of these measures ranged from 1 to 6, and it was anchored at every point (e.g., 1 = Strongly disagree, 6 = Strongly agree). Unlike risk perception and response efficacy, the renters had a much lower mean result, consistent with the practical reality of being a renter, as opposed to a buyer. We averaged the two items to create a self-efficacy scale. (Study 1: *r*_SB_ = 0.67, *M* = 3.18 *SD* = 1.36; Study 2: *r*_SB_ = 0.61, *M* = 4.61, *SD* = 1.12; Study 3: *r*_SB_ = 0.70, *M* = 4.72, *SD* = 1.19).

#### 2.3.5. Perceived Affordability

We included three items (e.g., “I could afford to spend $1000–$5000 replacing the lead pipes with lead-free ones in the new home”) to assess how affordable our participants believed that the replacement of lead pipes would be [25]. The response scale of this measure ranged from 1 to 6, and it was anchored at every point (e.g., 1 = Strongly disagree, 6 = Strongly agree). We averaged the items to create a perceived affordability scale. As with self-efficacy, the renters had a much lower mean result. (Study 1: *α* = 0.78, *M* = 2.69, *SD* = 1.31; Study 2: *α* = 0.76, *M* = 4.39, *SD* = 1.20; Study 3: *α* = 0.73, *M* = 4.50, *SD* = 1.13).

#### 2.3.6. Click on the Link for Additional Information about Lead

We tracked whether our participants clicked on the link to the online pamphlet. This resulted in a dichotomous variable, where 0 = did not click and 1 = clicked. In Study 1, 16% of the participants clicked on the link, in Study 2, it was 14.8%, and in Study 3, it was 15.4%.

### 2.4. Ethical Statement

All subjects gave their informed consent for their inclusion before they participated in the study. The study was conducted in accordance with the Declaration of Helsinki, and the protocol was approved by the Institutional Review Board of Cornell University (Protocol #1803007884).

## 3. Results

### 3.1. Overall Willingness to Engage in Each of the Renter or Buyer Behaviors 

For all statistical analyses, we used the software package SPSS Statistics 25.0 (IBM, Armonk, NY, USA). Datasets and syntaxes are available as supplements (see Supplementary Files S4–S9). Before reporting on the differences by randomized conditions within each scenario, we compared the respondents’ relative willingness to adopt the different risk mitigation behaviors described within each study. To do so, we conducted a one-way repeated measures analysis of variance (ANOVA), controlling for experimental conditions, to compare the ratings of willingness to adopt different responses to the presence of LSLs. We used Greenhouse–Geisser corrections when the assumption of sphericity was violated, and Bonferroni corrections for post hoc comparisons [26]. We present these within-study comparisons of strategies in Table 2.

In Study 1, featuring landlord disclosures to home renters, participants showed the greatest intentions to adopt RB1 (*M* = 4.46, *SD* = 1.52) and RB5 (*M* = 4.27, *SD* = 1.49), and the least intentions to adopt RB2 (*M* = 2.25, *SD* = 1.50) and RB4 (*M* = 2.67, *SD* = 1.64), with intentions to adopt RB3 (*M* = 3.89, *SD* = 1.69) in the middle. Controlling for the experimental conditions, the results of a one-way repeated measures ANOVA with Greenhouse–Geisser correction showed that the five renter behaviors (RBs) differed in the respondents’ intention levels to adopt each RB, *F*(2.93, 1944.71) = 51.46, *p* < 0.001, $\eta^{2}$ = 0.058. Post hoc tests with Bonferroni corrections showed that RB1 was significantly higher than RB2, RB3, RB4, and RB5, *ps* < 0.05. In addition, RB3 was significantly higher than RB2, and RB4, *ps* < 0.001. Also, RB4 was significantly higher than RB2, *p* < 0.001. Finally, RB5 was significantly higher than RB2, RB3, and RB4, *ps* < 0.01. Hence, asking the landlord to replace the LSL was the highest rated behavior, followed by looking for another home. Paying for replacement oneself was the lowest rated option. The results of the one-way repeated measures ANOVAs for all three studies are shown in Table 2.

In Study 2, featuring seller disclosures to home buyers, participants showed the most intention to adopt BB2 (*M* = 4.89, *SD* = 1.27) and BB3 (*M* = 4.88, *SD* = 1.30), and the least intention to adopt BB6 (*M* = 1.84, *SD* = 1.32), with intentions to adopt BB1 (*M* = 3.70, *SD* = 1.69), BB4 (*M* = 3.33, *SD* = 1.77), BB5 (*M* = 3.09, *SD* = 1.74), and BB7 (*M* = 3.87, *SD* = 1.55) in the middle. Controlling for the experimental conditions, the results of a one-way repeated measure ANOVA with Greenhouse–Geisser correction showed that the seven BBs differed in the respondents’ intention levels to adopt each BB, *F*(4.46, 2959.69) = 103.75, *p* < 0.001, $\eta^{2}$ = 0.110. Posthoc tests with Bonferroni corrections showed that BB1 was significantly higher than BB4, BB5, and BB6, *ps* < 0.001. BB2 was significantly higher than BB1, BB4, BB5, BB6, and BB7, *ps* < 0.001. BB3 was significantly higher than BB1, BB4, BB5, BB6, and BB7, *ps* < 0.001. BB4 was significantly higher than BB6, *p* < 0.001. BB5 was significantly higher than BB6, *p* < 0.001. BB7 was significantly higher than BB4, BB5, and BB6, *ps* < 0.001. Having the seller replace the LSL, or self-replacing it after deducting the cost from the sale price were the highest rated options. Doing nothing was the lowest rated option.

In Study 3, featuring home inspector disclosures to home buyers, participants showed the greatest intentions to adopt BB2 (*M* = 4.74, *SD* = 1.34), and BB3 (*M* = 4.79, *SD* = 1.36), and the least intentions to adopt BB5 (*M* = 2.79, *SD* = 1.69), and BB6 (*M* = 1.61, *SD* = 1.19), with intentions to adopt BB1 (*M* = 3.54, *SD* = 1.70), BB4 (*M* = 3.53, *SD* = 1.69), and BB7 (*M* = 3.65, *SD* = 1.64) in the middle. Controlling for experimental conditions, the results of a one-way repeated measures ANOVA with Greenhouse–Geisser correction showed that the seven buyer behaviors (BBs) differed in their intention levels to adopt each BB, *F*(4.78, 4140.64) = 142.51, *p* < 0.001, $\eta^{2}$ = 0.114. Posthoc tests with Bonferroni corrections showed that BB1 was significantly higher than BB5 and BB6, *ps* < 0.001. BB2 was significantly higher than BB1, BB4, BB5, BB6, and BB7, *p* < 0.001. BB3 was significantly higher than BB1, BB4, BB5, BB6, and BB7, *ps* < 0.001. BB4 was significantly higher than BB5 and BB6, *ps* < 0.001. BB5 was significantly higher than BB6, *p* < 0.001. BB7 was significantly higher than BB5 and BB6, *ps* < 0.001. As in Study 2, having the seller replace the LSL, or self-replacing it after deducting the cost from the sale price were the highest rated options. Doing nothing was the lowest rated option.

We organized the presentation of the results for the randomized disclosure conditions within these three studies in the sections that follow, this time reporting on all of the outcomes for the different conditions as a set. We used one-way ANOVAs featuring the three (in Studies 1 and 2) or four (in Study 3) randomized disclosure conditions as the independent variables, and the willingness to adopt risk mitigation behaviors, risk perceptions, response efficacy, self-efficacy, and affordability as the dependent variables, respectively. We again used Bonferroni corrections for post hoc comparisons. In addition, since “click on the link” was a binary variable, we used logistic regression featuring the three or four randomized conditions as the independent variables (one condition dummy-coded and omitted as the referent group), and “click on the link” as the dependent variable. 

### 3.2. Study 1: Landlord Disclosures to Home Renters

**Figure 2:**
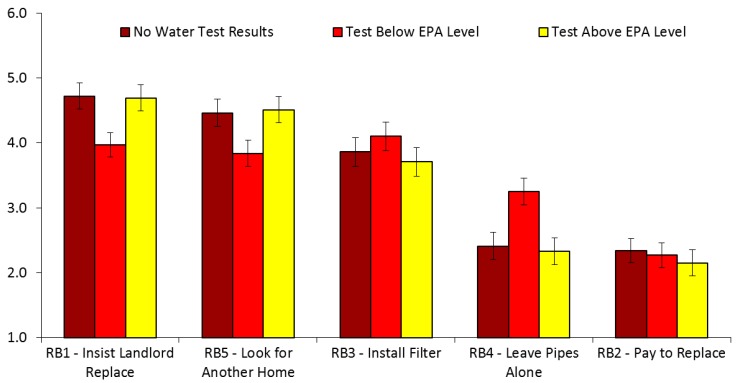
Willingness to adopt risk mitigation behaviors, by landlord/renter condition. Error bars indicate 95% confidence intervals with Bonferroni corrections.

We continued by comparing all study outcomes by randomized disclosure conditions (“No Water Test Results”, “Above EPA Level”, and “Below EPA Level”) in the landlord/renter study. ANOVAs and subsequent means comparisons (with Bonferroni corrections) revealed a number of statistically significant differences in willingness to take action to mitigate the risks associated with the presence of LSLs (see Figure 2 for visual display of these findings). First, there were significant differences for the willingness to insist on landlord replacement, *F*(2, 664) = 18.47, *p* < 0.001, $\eta^{2}$ = 0.053, and look for another home, *F*(2, 664) = 14.55, *p* < 0.001, $\eta^{2}$ = 0.042. Specifically, exposure to the “No Water Test Results” and the “Above EPA Level” conditions led to a greater willingness to insist on landlord replacement, and to look for another home, than did the “Below EPA Level” condition, *p*s < 0.001. These results are consistent with our hypothesis that the “Below EPA level” condition would produce lower levels of willingness to replace the LSL than the other two conditions. In addition, we observed the reverse pattern of results for the willingness to leave LSLs alone, *F*(2, 664) = 23.05, *p* < 0.001, $\eta^{2}$ = 0.065. In other words, the “No Water Test Results” and the “Above EPA Level” conditions did not differ from one another, and led to a lower level of willingness to leave the lead pipes alone, than did the “Below EPA Level” condition, *p*s < 0.001. We also found a significant difference for willingness to install and maintain a filter, *F*(2, 664) = 3.07, *p* = 0.047, $\eta^{2}$ = 0.009. In particular, the “Below EPA Level” condition produced a stronger willingness than the “Above EPA Level” condition, *p* = 0.043. 

We observed a similar pattern of results for risk perceptions, *F*(2, 664) = 4.72, *p* = 0.009, $\eta^{2}$ = 0.014. That is, the “No Water Test Results” and the “Above EPA Level” conditions led to higher risk perceptions than did the “Below EPA Level” condition, *p*s < 0.05. In an unexpected finding, the “Below EPA Level” condition led to more clicks on the lead pamphlet than the “Above EPA Level” condition, B = −0.62, SE = 0.27, *p* = 0.022. There were no other statistically significant differences by randomized experimental group for the other outcomes, as shown in Table 3. 

Overall, we note from Table 3 that Study 1 respondents across all conditions reported a high level of willingness to either (a) insist on landlord replacement of the LSL as a condition of renting, or to (b) look for another home to rent (with means for both items above the midpoint of 3.5 across all conditions). Risk perceptions and response efficacy were also very high across all three conditions (with means near or above 5 on a 6-point scale anchored at 5 with “agree”, and at 6 with “strongly agree”). The perceived self-efficacy and affordability were much lower, and less than one in four respondents clicked on the link for additional information about lead in any condition.

### 3.3. Study 2: Seller Disclosures to Home Buyers

**Figure 3:**
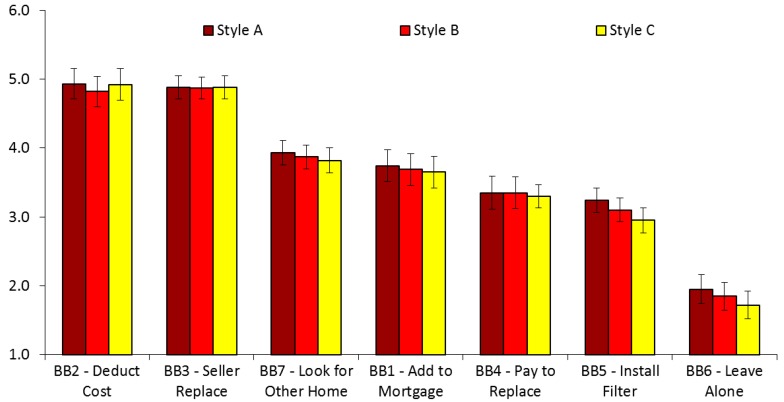
Willingness to adopt risk mitigation behaviors, by seller/buyer condition. Error bars indicate 95% confidence intervals, with Bonferroni corrections.

Next, we compared all of the study outcomes by randomized disclosure conditions (“Style A”, “Style B”, and “Style C”) in the seller/buyer study. ANOVAs revealed no statistically significant effects of any randomized experimental conditions on any of the dependent variables in Study 2. Regardless of the style of the disclosure, participants showed clear preferences for being willing to either deduct the cost of replacement from the price of the home, or to insist that the seller replace the LSL as a condition of the sale. These preferences were quite strong, and they corresponded to high values on this scale (closest to 5, anchored with “very likely”—see Figure 3). In contrast, very few respondents reported a willingness to leave the LSL alone, with the other options falling somewhere in the middle. 

Table 4 shows the mean levels of responses to each dependent variable, by condition. Across the experimental conditions, participants rated highly on risk perceptions and response efficacy, the means of which are all above 5 on a 1 to 6 scale. The perceived self-efficacy and affordability were slightly lower, but still well above the midpoint of the response scale. Fewer than one in six respondents clicked on the link to additional information about lead in any condition.

### 3.4. Study 3: Inspector Disclosures to Home Buyers

We continued by comparing all of the study outcomes by randomized disclosure conditions (Recommendations Present/Absent and Why Information Present/Absent) in the inspector/buyer study. In light of the fact that, as noted above, we did not see any significant differences between the three experimental conditions in the home seller/buyer study (Study 2) on any outcome, we chose to combine the three conditions, and to include them, as a whole, as a “comparison group” in the analyses for the home inspector/buyer study (Study 3). While it is true that the stimuli used for the two home buyer experiments were not the same, and they featured different sources of disclosure, we reason that the minimum level of disclosure information that was provided in the home seller/buyer experiment makes it a good standard of comparison to serve as a control group in which the participants knew about the presence of LSLs but nothing more.

Using one-way ANOVAs with Bonferroni corrections and logistic regression for the dichotomous outcome (clicking on the link), we examined whether exposure to any of the four inspector/buyer conditions produced any differences in any of the dependent variables, relative to (a) one another, or (b) the aggregated seller/buyer conditions serving as the control group. 

**Figure 4:**
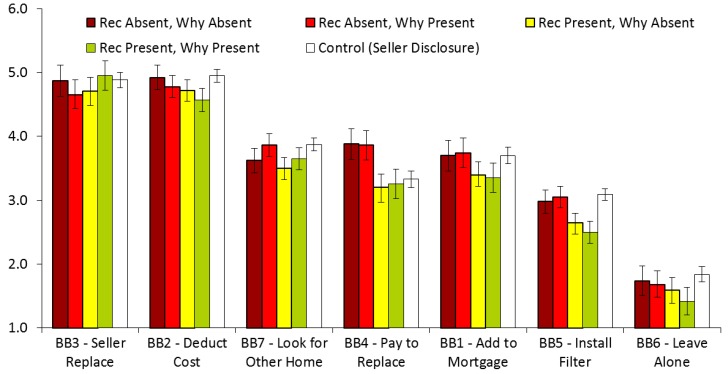
Willingness to adopt risk mitigation behaviors, by inspector/buyer condition. Error bars indicate 95% confidence intervals with Bonferroni corrections.

There were a number of statistically significant differences in the willingness to adopt specific risk mitigation strategies (Figure 4). First, we found a significant difference for the willingness to deduct the cost of LSL replacement from the sale price between the four conditions and the control, *F*(4, 1533) = 3.05, *p* = 0.016, $\eta^{2}$ = 0.008. Specifically, the willingness was lower when the inspector report contained an explicit recommendation and “why” information, relative to exposure to the seller disclosure control, *p* = 0.023. These results run contrary to *a priori* hypotheses, in which we expected the explicit recommendations and the “why” information to increase the willingness to have the LSL removed in the home-buying process. On the flipside, however, we observed the same between-conditions pattern for willingness to leave the LSLs alone, *F*(4, 1533) = 5.27, *p* < 0.001, $\eta^{2}$ = 0.014. That is, the willingness was lower when the inspector report contained an explicit recommendation and “why” information, relative to the exposure to the seller disclosure control group, *p* < 0.001.

Second, we found a significant difference for willingness to pay to replace the LSL by participants themselves, *F*(4, 1533) = 9.08, *p* < 0.001, $\eta^{2}$ = 0.023. In particular, participants were more willing to replace the LSL themselves when there were no explicit recommendations, regardless of the presence or absence of the “why” information, compared to the two conditions where the recommendations were present, or the control condition, *p*s < 0.01. 

Third, there was also a significant difference in willingness to install and maintain a filter, *F*(4, 1533) = 6.91, *p* < 0.001, $\eta^{2}$ = 0.018. Specifically, compared to when both a recommendation and “why” information was present, the two conditions without the recommendations and the control conditions produced more willingness among the participants, *p*s < 0.05. In addition, willingness was higher for the control condition than for the condition where recommendations were present, but the “why” information was absent, *p* = 0.005. Finally, we found a significant difference in willingness to look for another home, *F*(4, 1533) = 3.20, *p* = 0.013, $\eta^{2}$ = 0.008. Participants in the control condition were more willing to look for another home than those in the condition where recommendations were present, but the “why” information was absent, *p* = 0.019.

Table 5 shows the mean levels of responses to each dependent variable, by condition in the inspector/buyer study, as well as by the aggregate results from the seller/buyer study. Similar to what we found in the home seller/buyer study, regardless of the randomized experimental condition, participants showed a relatively high level of willingness to deduct the cost of the LSL replacement from the mortgage, or to insist that the seller replace the LSL as a condition of the sale. As for other measured variables, participants rated high on risk perceptions and response efficacy, though these variables did not differ by randomized condition, or between the inspector/buyer study conditions and the aggregate seller/buyer study conditions. Similarly, participants rated well above the scale midpoint on self-efficacy and affordability, but these values also did not differ by condition. Less than one in five respondents clicked on the pdf to learn about lead risks in any of the randomized conditions in the inspector/buyer study, or the aggregate seller/buyer study group.

## 4. Discussion

### 4.1. Summary of Findings and Interpretation of Results

Across three different home-buying or home-renting scenarios, and across disclosure conditions within these scenarios, respondents who received information about the presence of an LSL in a potential home reported a high level of willingness to adopt behaviors that would require the seller or the landlord to replace the LSL. In the renter study, across experimental manipulations, respondents reported being the most willing to ask the landlord to replace the LSL, followed by looking for another home. Paying for replacement oneself was the lowest rated option. In the two studies where we placed respondents in a home-buying scenario, respondents reported being most willing to ask the seller to replace the LSL, or replace it themselves after deducting the cost from the sale price. Leaving it alone after moving in was consistently the least preferred option in response to any form of disclosure. In addition, study participants reported generally high levels of perceived health risk of lead exposure, and strong beliefs that there were other effective steps that a potential homebuyer or renter could take, short of having the seller or landlord replace the LSL, to address the presence of an LSL in the home (i.e., response efficacy). Table 6 presents the key findings of the three experiments.

Based on these overall results, we conclude that home renters and home buyers who received information about LSLs appeared to clearly understand the importance of replacing them and avoiding homes with LSLs. Respondents were willing to push for LSL replacement by the current owners (or landlord), if the option existed. They were less enthusiastic about incurring the cost of replacement themselves, preferring to seek other properties to buy or to rent. 

Turning to the effects of various methods of disclosure, among potential home renters, we found that a landlord giving a potential renter water test results showing the lead level was below EPA’s lead action level appeared to give more people license to do less to replace the LSLs, as well as a false sense of security (as indicated by weaker risk perceptions), in contrast to results showing that the lead level was above the EPA’s action level, or providing no water test results at all. This finding is consistent with a previous study showing that providing basic risk information plus additional alarming information led to higher risk perceptions than basic risk information plus reassuring information [20]. These results suggest that there is a critical need to educate consumers about (a) the challenges and uncertainties involved in water test results, (b) their potential for a disproportionate influence in renter decision making, and (c) the fact that there is no safe level of lead exposure. 

Among potential home buyers, we found no difference in the three styles by which a seller would communicate the presence of an LSL to a potential homebuyer in a typical state-required disclosure form. Those styles were “Is lead plumbing present?”; “Are there any lead hazards? (e.g., lead paint, lead pipes, lead in soil.)”; or “Are you aware of any substances, materials, or products that may be an environmental hazard such as asbestos, formaldehyde, radon gas, lead-based paint, fuel or chemical storage tanks, contaminated soil, water or by-products from the production of methamphetamines on the subject property?” None of the three styles of communication influenced any key outcomes, including the willingness to take action, or risk or efficacy beliefs. The manner of a seller’s disclosure appears to make little difference, at least when the potential buyer is given basic information about the potential for a lead-related risk.

However, it is necessary for the seller to become aware of the presence of the LSL, and actually disclose it. Disclosure requirements that allow the seller to judge whether the LSL poses an environmental hazard are fraught with problems, given the incentives for the seller not to disclose. In addition, a seller’s willingness to disclose may be impacted if the seller suspects that the buyer will be able to learn about the LSL from another source, such as from an online search engine or map, a vigilant home inspector, or a welcome letter from the drinking water utility to the buyer as a new customer that highlights the LSL. Future work should explore the effects of these potential influences on the willingness of home sellers to search for information about the potential for an LSL to exist on their property, and/or to disclose its presence in the home selling process. 

We found that when the home buyer is given basic information about the risks posed by LSLs and the options to remove it, it can be counterproductive for the home inspector to recommend replacements or to explain why. This suggests that the cause for this may simply be that the home inspector is the person checking for the LSL and disclosing its presence (especially when the seller may not). In some cases, providing specific recommendations appeared to backfire, reducing willingness to pay to replace the LSL out of pocket or to install a lead filter to reduce potential lead exposure from the LSL. One possible explanation for this finding is the perceived motivation of the messenger making the disclosure. When home-sellers disclose a potential health risk to buyers, the disclosure may be perceived as an act of honesty or goodwill, which makes the recipient of the message, the potential buyers, heed the information and potentially form a favorable opinion of the seller and the seller’s suggestions. However, disclosure by a home inspector may not benefit from this perceived honesty or altruism. For many people, a disclosure from the inspector, especially one with a recommendation, may be perceived as an attempt to secure more business or work (via searching for and having to inspect additional potential homes), though inspectors would not be the ones carrying out the replacement. The respondent may not have been aware that the industry ethical code makes clear that a home inspector cannot do the work, because it undermines her or his credibility [27]. This information would be included in a typical inspection report, but it was not provided in our study to the respondent. Likewise, the respondents may see recommendations from a home inspector as being overly cautious, and intended to cover any potential liability related to not fully warning a potential homebuyer of the relevant risks. 

Another reason for the counterintuitive results may be that although the “why” information included more explicit information about the negative health impacts of lead on humans and pets, it failed to create greater risk perceptions about LSLs, and intentions to act. Respondents might not know of the exact consequences of exposure to lead, but may have been well-aware that exposure to lead is harmful to health, which overrode the impact of the “why” information. It is also possible that our participants were overwhelmed by the list of potential actions that they could take, especially when all of recommended actions required multiple steps to complete, and became, ironically, less motivated to take any of these actions [28]. Note that whenever explicit recommendations were mentioned, we also included a sentence with all letters capitalized, “WE STRONGLY RECOMMEND THAT YOU REPLACE THE LSL WITH A NON-LEAD PIPE”. This approach might have threatened our participants’ freedom to choose, which could potentially have increased psychological reactance [29].

In addition, we note that the Flint water crisis made national news only three years ago [30]. Public awareness about lead may have been particularly high during this moment, but may have waned over time. If this is true, then the manner in which the disclosure is communicated may make a difference in the future, and consumers may need additional information about the risks of lead exposure at that time. Another factor to consider is that our scenarios, unlike real life home-buying experiences, did not involve necessary repairs in the home that had to be negotiated with the home-sellers (although we did include reference to the need to replace energy inefficient windows at a similar cost). It may be that the style of disclosure of LSLs could matter when, for instance, there are multiple other issues with a home, but LSLs are the only ones that are directly related to a health risk.

### 4.2. Study Limitations

The main limitation of this study is its inability to assess the extent to which the examined variations in disclosure practices motivated actual behavior to remove an LSL. We relied on a series of hypothetical scenarios in which we asked respondents about their willingness to engage in a variety of behaviors upon the receipt of information about the presence of an LSL, but real-world home renting and home buying situations are much more complex, and they involve real investments of time and money. As with most studies, the mindset induced may not be the one in which people find themselves in the real world. In addition, because we recruited our respondents from a national sample, we expect that respondents may face different local structural and environmental constraints. For instance, the option of looking for another home may depend on how tight the local real estate market is. We also did not consider motivational factors such as the presence of a young child, a pregnant mother, or other at-risk populations in the home. These structural and individual factors are impossible to replicate in a single, controlled study. Further field research comparing actions by property owners to remove LSLs in a home that they wish to buy or rent in locales or states with different guidelines for LSL disclosure would be a useful complement to the findings presented here. 

Further, this study did not test how participants would react if they did not know that there was an LSL in the property in question. We designed the study to assess the impact of various forms of LSL disclosure, and not to compare home renter or home buyer reactions to the omission of information about LSLs. If people are not told that a home has an LSL, or even of the existence of LSLs, they have no real opportunity to replace it or to otherwise reduce their risk of lead exposure; for example, by purchasing a water filter, or by searching for another home to rent or purchase. Indeed, in most states, the disclosure of LSLs is not mandatory. A hypothetical scenario is not very well-equipped to test this, however, as suggested above. This question should be addressed by further observational field research that, for example, compares the rates of LSL replacement in cities or states that mandate disclosure, versus ones that do not.

The dollar range that we used to estimate private-side LSL replacement ($1000–$5000) may be low, as there is no robust data providing a precise average range for this work across the U.S. If it is low, we may have observed a higher willingness to take action compared to that in the real world.

Lastly, the sample was skewed toward white, middle-class respondents, so it may not represent the response from a lower income or minority resident sample. This audience may have more latitude than minority or low-income individuals to negotiate with a seller or landlord. More broadly, M-Turk samples have been criticized for their broader concerns about generalizability, although recent evidence suggests that randomized experimental studies may produce similar conclusions about the effects of messages on cognitions and behavior [31]. 

### 4.3. Preliminary Recommendations for LSL Disclosure Policy and Practice

Despite these limitations, study results demonstrate that homebuyers and renters are concerned about LSLs, and that they appear willing to take steps to address the issue if they learn that an LSL is present. We conclude with a few preliminary recommendations for effective disclosure. 

#### 4.3.1. Requiring LSL Disclosure

Currently there is no federal disclosure requirement for LSLs and few relevant state requirements. None of the state laws specifically addresses LSLs, but rather “lead pipes” or “lead plumbing.” State policies could generally be improved by specifically (a) mandating that property owners disclose LSLs to potential homebuyers or renters, whether as a material used in plumbing or as an example of an environmental hazard; and (b) requiring drinking water utilities to notify property owners of the likely presence of an LSL, and making that information publicly available. This approach would avoid the market breakdown that occurred with federal lead-based paint disclosure, by ensuring the property owner has knowledge of the LSL—knowledge which the current study suggests is likely to motivate concern and willingness to take action among potential home buyers and renters. This would give the property owner an additional incentive to disclose, in order to avoid potential liability if a homebuyer first learns about the LSL through a “new customer” letter from the water utility. In addition, this would enable home inspectors to have access to the information, so they can inform the potential homebuyer. We make this preliminary recommendation with the recognition that once the presence of an LSL is disclosed, the cost of replacement may place a disproportionate burden on already disadvantaged communities. Policymakers should consider these inequalities when developing policies and allocating resources, to support widespread LSL disclosure and replacement.

#### 4.3.2. Ensuring that Inspectors Look for LSLs

The current study suggests that inspectors could play an important role in disclosure. The American Society of Home Inspectors (ASHI) Standard of Practice for Home Inspectors requires inspection and description of the pipe material of the interior water supply and distribution system, which presumably would include the portion of the service line as it enters the building [27]. We recommend that ASHI or other organizations, such as the International Association of Certified Home Inspectors, include in their inspector training curriculum a segment on identifying an LSL. While providing buyers with explicit recommendations for addressing LSLs may not be critical, buyers need to understand the specific options that they have at their disposal to remove the LSL, or to otherwise reduce their risk from lead in water. By virtue of taking the survey, all respondents in our study were presented with such options, and when given such options, they frequently expressed a willingness to take actions that would remove the LSL entirely.

#### 4.3.3. Dissociating Water Testing Results from LSL Disclosure

Water testing for lead is unpredictable—with results varying based on water chemistry, temperature, and disturbances to the plumbing—and they are unlikely to determine whether or not an LSL is present. Lead in water testing is even more complex in multifamily housing, where many renters live. However, we found that if people are told that a one-time water test shows lead levels are less than EPA’s lead action level of 15 ppb, they reported as being less willing to take action on an LSL (in the absence of full information about the limitations of the one-time test, and that there is no safe exposure to lead). We only tested this in the renter situation, but it seems likely that these findings would apply to buyers, too. Given this risk, we recommend that LSL disclosure not be linked too closely with water testing results, as it may decrease the likelihood of the potential resident taking action to address the LSL. At a minimum, regulatory agencies could require that all water test results be disclosed, not just those that serve the landlord or seller’s interests, and that they are accompanied by a statement that explains the limitations of testing, and that a risk is present as long as there is an LSL present. 

## 5. Conclusions

These three studies evaluated the effectiveness of disclosure styles in influencing respondents’ perceived risks of having an LSL in a home, and their willingness to act if they did have an LSL. When the scenarios were about renting, having landlords disclose the presence of an LSL and provide water test results that showed lead levels below the recommended action levels resulted in lower levels of perceived risk, and of a willingness to act, relative to renting scenarios where the water test results showed lead levels above the recommended action levels, or relative to scenarios with no water test results. Based on this finding, it seems wise that LSL disclosures not be linked with water testing results, because the latter may decrease motivation among renters to take action. In seller-disclosure home buying scenarios, the levels of perceived risk and the willingness to act were consistently high, with no statistically significant variation by disclosure styles on any outcome variables. We interpret this as showing support for policies that require the disclosure of the presence of LSLs, both by the sellers and the public utilities, since the latter are responsible for the public side of the service line. Finally, our home inspector-disclosure scenarios produced levels of perceived risk and willingness to act that were quite high. Explicit recommendations to replace the service lines, and the presence of information about risk did not further influence the risk perception or the willingness to ask, and for some outcome variables, they actually led to lower levels of intent to act. These data suggest that LSL disclosures by home inspectors could be useful practices for protecting families from the risks posed by LSLs. However, while the study showed that the provision of explicit recommendations in the inspector reports was not effective, when faced with the plethora of decisions that emerge in the home buying process, buyers do need a source of information about the available methods for removing LSLs, or for reducing the levels of lead in water. Field studies of these disclosures are necessary, to grasp the effectiveness of all the disclosure styles and circumstances that are simulated in the studies.

## Figures and Tables

**Table 1:** Participant demographics.

Demographics	Landlord/Renter	Seller/Buyer	Inspector/Buyer
	*N* = 667	*N* = 667	*N* = 871
**Age** (mean)	33.3	42.1	42.0
**Gender** (%)			
Male	47.1	42.1	40.3
Female	51.3	57.1	59.4
Transgender or other category	0.6	0.6	0.3
**Education** (%)			
Some high school or less	0.5	0.5	0.6
High school diploma/equivalent	10.0	7.9	8.2
Some college, no degree	25.5	17.1	18.4
Associate degree	12.3	12.0	14.5
Bachelor’s degree	37.5	42.9	40.6
Master’s degree	10.8	15.7	14.2
Professional degree (MD, JD)	2.1	2.1	2.3
Doctorate degree	1.2	1.6	1.3
**Race** (%)			
White	80.4	86.4	85.4
Black or African American	11.4	7.3	7.6
American Indian/Alaska Native	2.5	1.8	2.0
Asian/Indian	2.1	1.3	2.1
Chinese, Japanese or Korean	3.8	1.8	3.1
Filipino or Vietnamese	2.1	1.6	1.4
Pacific Islander/Hawaii Native	0.1	0	0.4
Other Asian	1.2	0.6	0.5
Other race	2.8	1.8	1.4
**Hispanic/Latino** (%)	11.5	7.5	8.4
**Marital status** (%)			
Married	29.1	56.1	59.5
Widowed	0.9	2.4	1.6
Divorced	4.8	10.8	10.1
Separated	1.3	1.6	1.4
Never married	42.7	18.0	16.2
Living with a partner	21.1	11.1	11.3
**Household income** (%)			
$24,999 or less	21.9	9.4	8.5
$25,000 to $34,999	19.2	10.2	8.7
$35,000 to $49,999	20.4	16.8	13.9
$50,000 to $74,999	23.7	24.7	24.9
$75,000 to $99,999	9.3	18.1	22.3
$100,000 to $149,999	3.6	12.9	15.7
$150,000 or more	1.9	7.8	6.0
**Own home** (%)	8.2	73.3	75.2
**Have children** (%)	30.7	60.7	61.4
**Living with children under 12** (%)	25.9	34.6	37.4
**Have pets** (%)	58.0	68.8	71.2
**Political party** (%)			
Republican	13.3	28.5	26.4
Democrat	46.3	39.1	37.8
Independent	32.1	26.5	29.5
Another party	2.5	1.3	1.1
No preference	5.7	4.5	5.2
**Political ideology** (%)			
Liberal	60.3	48.7	45.9
Moderate	18.7	18.4	20.6
Conservative	20.9	33.8	33.6
**Heard of LSLs before** (%)	18.6	29.8	29.9

LSLs: Lead service lines.

**Table 2:** Differences in respondents’ willingness to engage in responses to the presence of LSLs by study, collapsing across randomized conditions.

Specific Renter Behavior (RB) or Buyer Behavior (BB)	Study 1	Study 2	Study 3
	*N* = 667	*N* = 667	*N* = 871
	*M* (*SE*)	*M* (*SE*)	*M* (*SE*)
**BB1**: I would add the cost of replacement ($1000–$5000) to the mortgage and replace the lead pipes after purchasing the home, but before moving in.	N/A	3.70 (0.07) ^a,b,c,d,e^	3.54 (0.06) ^a,b,c,d^
**BB2**: I would deduct the estimated cost ($1000–$5000) of replacing the lead pipes from the sale price and use those funds to replace the pipes before moving in.	N/A	4.89 (0.05) ^a,f,g,h,i^	4.74 (0.05) ^a,e,f,g,h^
**RB1/BB3**: I would insist that the (landlord/seller) replace the lead pipes with non-lead pipes (as a condition of renting/prior to closing on the home).	4.46 (0.06) ^a,b,c,d^	4.88 (0.05) ^b,j,k,l,m^	4.79 (0.05) ^b,i,j,k,l^
**RB2/BB4**: I would pay to replace the lead pipes ($1000–$5000) after moving in.	2.25 (0.06) ^a,e,f,g^	3.33 (0.07) ^c,f,j,n,o^	3.53 (0.06) ^e,i,m,n^
**RB3/BB5**: I would move in, and install and maintain a filter designed to remove lead even though I must replace the filter monthly at a cost of about $150 a year.	3.89 (0.07) ^b,e,h,i^	3.09 (0.07) ^d,g,k,p,q^	2.79 (0.06) ^c,f,j,m,o,p^
**RB4/BB6**: I would move in and leave the lead pipes alone.	2.67 (0.06) ^c,f,h,j^	1.84 (0.05) ^e,h,l,n,p,r^	1.61 (0.04) ^d,g,k,n,o,q^
**RB5/BB7**: I would look for another home to (rent/buy).	4.27 (0.06) ^d,g,i,j^	3.87 (0.06) ^i,m,o,q,r^	3.65 (0.06) ^h,l,p,q^

Table note: These variables were measured on a 1–6 scale. Cells with the same letter in the same column (i.e, ^a^, ^b^, ^c^, ^d^, ^e^, ^f^, ^g^, ^h^, ^i^, ^j^, ^k^, ^l^, ^m^, ^n^, ^o^, ^p^, ^q^, ^r^) in each panel are statistically different at *p* < 0.05 (One-way repeated measures ANOVA results with Bonferroni corrections). M: mean; SE: standard error.

**Table 3:** Mean differences between disclosure conditions in the landlord/renter study (willingness items sorted by mean willingness to adopt action within Study 1).

Outcome Variables	No Water Test Results	Below EPA Action Level	Above EPA Action Level
	*N* = 226	*N* = 225	*N* = 216
	*M* (*SD*)	*M* (*SD*)	*M* (*SD*)
**RB1**: I would insist that the landlord replace the lead pipes as a condition of renting.	4.72 (1.35) ^a^	3.97 (1.70) ^a,b^	4.69 (1.38) ^b^
**RB5**: I would look for another home to rent.	4.46 (1.33) ^a^	3.84 (1.56) ^a,b^	4.51 (1.49) ^b^
**RB3**: I would move in, and install and maintain a filter that is designed to remove lead, even though I must replace the filter monthly at a cost of about $150 a year.	3.86 (1.67)	4.10 (1.62) ^a^	3.71 (1.76) ^a^
**RB4**: I would move in and leave the lead pipes alone.	2.41 (1.51) ^a^	3.25 (1.72) ^a,b^	2.33 (1.54) ^b^
**RB2**: I would pay to replace the lead pipes ($1000–$5000) after moving in.	2.34 (1.57)	2.27 (1.48)	2.15 (1.43)
**Risk perceptions**	5.09 (0.74) ^a^	4.92 (0.70) ^a,b^	5.11 (0.75) ^b^
**Response efficacy**	5.15 (0.88)	5.16 (0.96)	5.16 (0.94)
**Self-efficacy**	3.25 (1.35)	3.13 (1.34)	3.15 (1.39)
**Affordability**	2.69 (1.30)	2.69 (1.30)	2.68 (1.32)
**Click on the pamphlet link (%)**	17%	20% ^a^	12% ^a^

Table note: Unless otherwise indicated, these variables were measured on a 1–6 scale. Conditions with the same letter in the same row (i.e., ^a^, ^b^) are statistically different, at *p* < 0.05 (One-way ANOVA results with Bonferroni corrections; the results for “click on the link” was based on a logistic regression). M: mean; SD: standard deviation.

**Table 4:** Mean differences between the disclosure conditions in the seller/buyer study (willingness items sorted by mean willingness to adopt action within Study 2).

Outcome Variables	Style A	Style B	Style C
	*N* = 214	*N* = 230	*N* = 223
	*M* (*SD*)	*M* (*SD*)	*M* (*SD*)
**BB2**: I would deduct the estimated cost ($1000–$5000) of replacing the lead pipes from the sale price, and use those funds to replace the pipes before moving in.	4.93 (1.21)	4.82 (1.32)	4.92 (1.27)
**BB3**: I would insist that the seller replace the lead pipes with non-lead pipes prior to closing on the home.	4.88 (1.30)	4.87 (1.37)	4.88 (1.24)
**BB7**: I would look for another home to buy.	3.93 (1.58)	3.87 (1.58)	3.82 (1.50)
**BB1**: I would add the cost of replacement ($1000–$5000) to the mortgage, and replace the lead pipes after purchasing the home but before moving in.	3.74 (1.69)	3.69 (1.66)	3.65 (1.72)
**BB4**: I would pay to replace the lead pipes ($1000–$5000) after moving in.	3.35 (1.75)	3.35 (1.77)	3.30 (1.80)
**BB5**: I would move in, and install and maintain a filter designed to remove lead, even though I must replace the filter monthly at a cost of about $150 a year.	3.24 (1.75)	3.10 (1.74)	2.95 (1.71)
**BB6**: I would move in and leave the lead pipes alone.	1.95 (1.41)	1.85 (1.33)	1.72 (1.22)
**Risk perceptions**	5.09 (0.82)	5.11 (0.81)	5.06 (0.75)
**Response efficacy**	5.28 (0.89)	5.26 (0.92)	5.18 (0.93)
**Self-efficacy**	4.59 (1.19)	4.58 (1.08)	4.67 (1.11)
**Affordability**	4.45 (1.18)	4.39 (1.17)	4.33 (1.25)
**Click on the link (%)**	15%	14%	15%

Table note: Unless otherwise indicated, these variables were measured on a 1–6 scale. Conditions with the same letter in the same row are statistically different at *p* < 0.05 (One-way ANOVA results with Bonferroni corrections; the result for “click on the link” was based on a logistic regression). M: mean; SD: standard deviation.

**Table 5:** Mean differences between disclosure conditions in the inspector/buyer study, and the aggregate results from the seller/buyer study (willingness items sorted by means).

Outcome Variables	Rec. Absent Why Absent	Rec. Absent Why Present	Rec. Present Why Absent	Rec. Present Why Present	Seller Disclosure
	*N* = 195	*N* = 214	*N* = 251	*N* = 211	*N* = 667
	*M* (*SD*)	*M* (*SD*)	*M* (*SD*)	*M* (*SD*)	*M* (*SD*)
**BB2**: Deduct from sale price	4.92 (1.28)	4.77 (1.33)	4.72 (1.29)	4.57 (1.45) ^a^	4.89 (1.27) ^a^
**BB3**: Insist seller replace	4.87 (1.29)	4.65 (1.36)	4.71 (1.41)	4.95 (1.35)	4.88 (1.30)
**BB4**: Pay to replace	3.88 (1.65) ^a,b,c^	3.86 (1.68) ^d,e,f^	3.20 (1.59) ^a,d^	3.26 (1.71) ^b,e^	3.33 (1.77) ^c,f^
**BB1**: Add cost to mortgage	3.70 (1.71)	3.74 (1.69)	3.39 (1.66)	3.35 (1.74)	3.70 (1.69)
**BB7**: Look for other home	3.62 (1.68)	3.86 (1.65)	3.50 (1.64) ^a^	3.65 (1.56)	3.87 (1.55) ^a^
**BB5**: Install filter	2.98 (1.69) ^a^	3.05 (1.70) ^b^	2.65 (1.70) ^c^	2.50 (1.61) ^a,b,d^	3.09 (1.74) ^c,d^
**BB6**: Leave pipes alone	1.74 (1.35)	1.68 (1.25)	1.59 (1.14)	1.42 (1.01) ^a^	1.84 (1.32) ^a^
**Risk perceptions**	5.15 (0.86)	5.21 (0.82)	5.08 (0.82)	5.32 (0.72)	5.09 (0.79)
**Response efficacy**	5.37 (0.82)	5.33 (0.91)	5.35 (0.90)	5.49 (0.80)	5.24 (0.91)
**Self-efficacy**	4.68 (1.27)	4.77 (1.14)	4.70 (1.14)	4.71 (1.22)	4.61 (1.12)
**Affordability**	4.56 (1.16)	4.57 (1.05)	4.42 (1.13)	4.46 (1.18)	4.39 (1.20)
**Click on the link (%)**	17%	17%	15%	13%	15%

Table Note: “Why” refers to conditions where additional information about why to replace an LSL was provided. “Rec” refers to conditions where explicit recommendations about strategies to ensure the removal of the LSL before moving into the home were provided. See Table 4 for the exact wording of the willingness to adopt risk mitigation behavior items. Unless otherwise indicated, all the variables were measured on a 1–6 scale. Conditions with the same letter (i.e., ^a^, ^b^, ^c^, ^d^, ^e^, ^f^) in the same row are statistically different at *p* < 0.05 (One-way ANOVA results with Bonferroni corrections; the result for “click on the link” was based on a logistic regression). M: mean; SD: standard deviation.

**Table 6:** Key findings.

▪ In home buying scenarios, levels of perceived risk and willingness to act were consistently high.
▪ In renting scenarios, providing water test results that showed lead levels that were below EPA’s action level of 15 ppb resulted in lower levels of perceived risk from LSLs, and of willingness to act.
▪ In seller-disclosure scenarios, three different disclosure styles did not differentially influence the levels of perceived risk or willingness to act.
▪ In inspector-disclosure scenarios, explicit recommendations to replace LSLs, and the presence of information about risk did not further influence the levels of perceived risk or willingness to act. In some cases, the inclusion of specific recommendations backfired.

## Supplementary Materials

The following are available online at https://www.mdpi.com/1660-4601/16/6/963/s1, File S1: Complete Text of LSL Disclosures and Full Questionnaire, Landlord/Renter Study; File S2: Complete Text of LSL Disclosures and Full Questionnaire, Seller/Buyer Study; File S3: Complete Text of LSL Disclosures and Full Questionnaire, Inspector/Buyer Study; File S4: Full Dataset, Landlord/Renter Study; File S5: Analysis Syntax, Landlord/Renter Study; File S6: Full Dataset, Seller/Buyer Study; File S7: Analysis Syntax, Seller/Buyer Study; File S8: Full Dataset, Inspector/Buyer Study with Seller/Buyer Conditions Added; File S9: Analysis Syntax, Inspector/Buyer Study with Seller/Buyer Conditions Added.
