# Supplementary material for: Research to Move Toward Evidence-Based Recommendations for Lead Service Line Disclosure Policies in Home Buying and Home Renting Scenarios
Source: Int J Environ Res Public Health. 2019 Mar 18;16(6):963. doi: 10.3390/ijerph16060963 (PMC6466339; doi:10.3390/ijerph16060963)
Supplement: Supplementary file 1 [file ijerph-16-00963-s001.zip › Supplementary files/Seller_Buyer Stimuli and Quesionnaire.pdf]

Writing Task

Imagine that you have been looking to **buy** a new home and have just found the right place—it is within your price range and fits all your needs. Please write 2-3 sentences to describe what your perfect home may look like.

Style A Condition

Imagine that you have already filed the paperwork and made an offer to **buy** the perfect home you just identified, and the seller has accepted your offer.

In the disclosure statement of certain conditions and information concerning the property, the seller checked the box below:

Is lead plumbing present?
Yes _X_ No ___ Unknown ___
If yes, state location or locations: *Lead service line connecting main under street to home*

Style B Condition

Imagine that you have already filed the paperwork and made an offer to **buy** the perfect home you just identified, and the seller has accepted your offer.

In the disclosure statement of certain conditions and information concerning the property, the seller checked the box below:

Are there any lead hazards? (e.g., lead paint, lead pipes, lead in soil.)
Yes _X_ No ___ Unknown ___
If yes, describe: *Lead service line connecting main under street to home*

Style C Condition

Imagine that you have already filed the paperwork and made an offer to **buy** the perfect home you just identified, and the seller has accepted your offer.

In the disclosure statement of certain conditions and information concerning the property, the seller checked the box below:

Are you aware of any substances, materials, or products that may be an environmental hazard such as asbestos, formaldehyde, radon gas, lead-based paint, fuel or chemical storage tanks, contaminated soil, water or by-products from the production of methamphetamines on the subject property?
Yes _X_ No ___ Unknown ___
If yes, describe: *Lead service line connecting main under street to home*

Link Seen by All Participants

As part of the sale, the seller is required to provide you with a 20-page pamphlet, “Protect Your Family from Lead in Your Home”, which can also be accessed at this web page: http://www.epa.gov/sites/production/files/2017-06/documents/pyf_color_landscape_format_2017_508.pdf. On page 13, you can find information on lead in drinking water. You may look at this information if you choose, or proceed to the next page of the survey.

Powered by Qualtrics

Intro

Thinking about this **home buying** scenario and the information you have been provided, please answer the questions on the following pages.

Questions Block 1

Please rate your likelihood of doing the following regarding the windows in the new home.

I would insist that the seller replace the single-pane windows as a condition of purchase.

Extremely unlikely	Moderately unlikely	Slightly unlikely	Slightly likely	Moderately likely	Extremely likely
○	○	○	○	○	○

I would pay ($1,000-$5,000) to replace the single-pane windows after moving in.

Extremely unlikely	Moderately unlikely	Slightly unlikely	Slightly likely	Moderately likely	Extremely likely
○	○	○	○	○	○

I would move in and leave the single-pane windows alone.

Extremely unlikely	Moderately unlikely	Slightly unlikely	Slightly likely	Moderately likely	Extremely likely
○	○	○	○	○	○

I would look for another home to buy.

Extremely unlikely	Moderately unlikely	Slightly unlikely	Slightly likely	Moderately likely	Extremely likely
○	○	○	○	○	○

Questions Block 2

Please rate your likelihood of doing the following regarding the lead service line in the new home.

I would add the cost of replacement ($1,000-$5,000) to the mortgage and replace the lead pipes after purchasing the home but before moving in.

Extremely unlikely	Moderately unlikely	Slightly unlikely	Slightly likely	Moderately likely	Extremely likely
O	O	O	O	O	O

I would deduct the estimated cost ($1,000-$5,000) of replacing the lead pipes from the sale price and use those funds to replace the pipes before moving in.

Extremely unlikely	Moderately unlikely	Slightly unlikely	Slightly likely	Moderately likely	Extremely likely
O	O	O	O	O	O

I would insist that the seller replace the lead pipes with non-lead pipes prior to closing on the home.

Extremely unlikely	Moderately unlikely	Slightly unlikely	Slightly likely	Moderately likely	Extremely likely
O	O	O	O	O	O

I would pay to replace the lead pipes ($1,000-$5,000) after moving in.

Extremely unlikely	Moderately unlikely	Slightly unlikely	Slightly likely	Moderately likely	Extremely likely
O	O	O	O	O	O

I would move in, and install and maintain a filter designed to remove lead even though I must replace the filter monthly at a cost of about $150 a year.

Extremely unlikely	Moderately unlikely	Slightly unlikely	Slightly likely	Moderately likely	Extremely likely
O	O	O	O	O	O

I would move in and leave the lead pipes alone.

Extremely unlikely	Moderately unlikely	Slightly unlikely	Slightly likely	Moderately likely	Extremely likely
O	O	O	O	O	O

I would look for another home to buy.

Extremely unlikely	Moderately unlikely	Slightly unlikely	Slightly likely	Moderately likely	Extremely likely
O	O	O	O	O	O

Questions Block 3

How likely would you be to do each of the following?

I would look for additional information about lead service lines.

Extremely unlikely	Moderately unlikely	Slightly unlikely	Slightly likely	Moderately likely	Extremely likely
O	O	O	O	O	O

I would share information about lead service lines with others.

Extremely unlikely	Moderately unlikely	Slightly unlikely	Slightly likely	Moderately likely	Extremely likely
O	O	O	O	O	O

I would avoid looking at information about lead service lines.

Extremely unlikely	Moderately unlikely	Slightly unlikely	Slightly likely	Moderately likely	Extremely likely
O	O	O	O	O	O

I would contact the water department to urge them to take action to replace lead service lines.

Extremely unlikely	Moderately unlikely	Slightly unlikely	Slightly likely	Moderately likely	Extremely likely
O	O	O	O	O	O

I would participate in a town hall meeting in support of action to replace lead service lines.

Extremely unlikely	Moderately unlikely	Slightly unlikely	Slightly likely	Moderately likely	Extremely likely
O	O	O	O	O	O

I would sign a petition in support of taking action to replace lead service lines.

Extremely unlikely	Moderately unlikely	Slightly unlikely	Slightly likely	Moderately likely	Extremely likely
O	O	O	O	O	O

I would join or volunteer with an organization working to replace lead service lines.

Extremely unlikely	Moderately unlikely	Slightly unlikely	Slightly likely	Moderately likely	Extremely likely
O	O	O	O	O	O

I would host a neighborhood meeting in your home to discuss actions people can take to replace lead service lines.

Extremely unlikely	Moderately unlikely	Slightly unlikely	Slightly likely	Moderately likely	Extremely likely
O	O	O	O	O	O

Questions Block 4

To what extent did you feel each of the following emotions when you were reading information on **lead service lines** in the inspector's report?

	None at all	Very slightly	A little	A moderate amount	A great deal	Extremely
Annoyed	○	○	○	○	○	○
Scared	○	○	○	○	○	○
Disgusted	○	○	○	○	○	○
Enthusiastic	○	○	○	○	○	○
Hopeful	○	○	○	○	○	○
Angry	○	○	○	○	○	○
Afraid	○	○	○	○	○	○
Optimistic	○	○	○	○	○	○
Sad	○	○	○	○	○	○

To what extent do you agree or disagree with the following statements?

	Strongly disagree	Disagree	Somewhat disagree	Somewhat agree	Agree	Strongly agree
Exposure to lead has serious negative consequences.	○	○	○	○	○	○
Lead exposure can harm children and lower their quality of life.	○	○	○	○	○	○
People in my home could be at risk for exposure to a high level of lead if my home had a lead service line.	○	○	○	○	○	○
Lead service lines in my home would expose me to lead in my drinking water.	○	○	○	○	○	○
Lead can result in heart disease, even at low levels.	○	○	○	○	○	○

To what extent do you agree or disagree with the following statements?

	Strongly disagree	Disagree	Somewhat disagree	Somewhat agree	Agree	Strongly agree
One way to significantly reduce exposure to lead in drinking water would be to replace the lead service line with a lead-free line.	○	○	○	○	○	○
I do not think it would be possible for me to replace the lead pipes in the new home with lead-free ones.	○	○	○	○	○	○
I could afford to spend $1,000-$5,000 replacing the lead pipes with lead-free ones in the new home.	○	○	○	○	○	○
I would be willing to spend $1,000–$5,000 to replace the lead pipes with lead-free ones in the new home.	○	○	○	○	○	○
	Strongly disagree	Disagree	Somewhat disagree	Somewhat agree	Agree	Strongly agree
I think it would be worth spending $1,000-$5,000 to replace the lead pipes with lead-free ones in the new home.	○	○	○	○	○	○
Having lead pipes replaced with lead-free ones would significantly reduce exposure to lead for people in the new home.	○	○	○	○	○	○
I would be able to have lead pipes replaced with lead-free ones in the new home.	○	○	○	○	○	○

Please estimate your knowledge of **lead service lines** on a 0-100 scale, where 0 means knowing nothing and 100 means knowing everything you could possibility know about the topic. How much do you think you currently know?

0 10 20 30 40 50 60 70 80 90 100

Think of that same scale again. This time, please estimate how much knowledge you would need to achieve an understanding of **lead service lines** that is good enough for your purposes. Of course, you might feel you need the same, more, or possibly even less, information about this topic. Using a scale of 0-100, how much information would be sufficient for you, that is, good enough for your purposes?

0 10 20 30 40 50 60 70 80 90 100

How likely would you be to go to the following sources to learn more about lead service lines?

	Extremely unlikely	Moderately unlikely	Slightly unlikely	Slightly likely	Moderately likely	Extremely likely
The Internet	○	○	○	○	○	○
Traditional media (e.g, TV, radio, newspaper, magazine)	○	○	○	○	○	○
My local, state, or federal government	○	○	○	○	○	○
The water utility company	○	○	○	○	○	○
Friends and family	○	○	○	○	○	○

What would you like to learn more about regarding lead service lines?

- ☐ How having a lead service line affects the value of a home
- ☐ The health effects of lead exposure
- ☐ The cost of replacing a lead service line

- ☐ How the replacement works
- ☐ Other (Please specify)
- ☐ I do not want to learn more about lead service lines.

Do you own the home where you live?

- ○ No, my housemate owns my home.
- ○ No, I rent my home.
- ○ Yes, I own or co-own my home.
- ○ Other

Do you know if your home (owned or rented) has a lead service line?

- ○ No, I do not know.
- ○ No, but I used to have one and it was removed.
- ○ Yes, I know that I do not have one.
- ○ Yes, I know that I have one.

When you signed the contract to buy your home, or the lease to rent your home, do you remember seeing a clause about whether or not lead plumbing or lead pipes were present?

- ○ No.
- ○ Yes.
- ○ I don't know.

If you discovered that your rental home had a lead service line on the private side, would you ask your landlord to replace it?

- ○ No.
- ○ Yes.
- ○ I don't know.

Do you think your landlord would comply with this request?

- No.
- Yes.
- I don't know.

How many years have passed since you bought the home where you live?

If you were to buy a new home within the next 5 years, what price range would you be looking for?

- $75,000 or less
- $75,001 - $125,000
- $125,001 - $175,000
- $175,001 - $225,000
- $225,001 - $300,000
- $300,001 - $500,000
- $500,001 or more

How do you describe yourself?

- Male
- Female
- Transgender man
- Transgender woman
- Do not identify as male, female, or transgender

In what year were you born?

What is your current residential zip code?

Are you now married, widowed, divorced, separated, never married, or living with a partner?

- Married
- Widowed
- Divorced
- Separated
- Never married
- Living with a partner

What is the highest level of school you have completed?

- Grade 8 or lower
- Some high school, no diploma
- High school diploma, or equivalent
- Some college, no degree
- Associate degree
- Bachelor's degree
- Master's degree
- Professional degree (MD, JD)
- Doctorate degree

Last year, before taxes, what would you say was your household income?

- $24,999 or less
- $25,000 to $34,999
- $35,000 to $49,999
- $50,000 to $74,999
- $75,000 to $99,999
- $100,000 to $149,999

- $150,000 to $199,999
- $200,000 or more

Are you Hispanic, Latino/a/x, or of Spanish origin?

- No, not of Hispanic, Latino/a/x, or Spanish origin
- Yes, Mexican American, Chicano
- Yes, Puerto Rican
- Yes, Cuban
- Yes, another Hispanic, Latino/a/x, or Spanish origin

What race or races do you consider yourself to be? Please select 1 or more of these categories.

- Black or African American
- White
- American Indian or Alaska Native
- Asian Indian
- Chinese
- Filipino
- Japanese
- Korean
- Vietnamese
- Native Hawaiian
- Guamanian or Chamorro
- Samoan
- Other Asian
- Other Pacific Islander
- Other

Generally speaking, do you think of yourself as a...?

- Republican
- Democrat
- Independent
- Another party
- No preference

If you had to choose, do you think of yourself as closer to the...?

- Republican party
- Democratic party

In general, do you think of yourself as...?

- Extremely liberal
- Liberal
- Slightly liberal
- Moderate, middle of the road
- Slightly conservative
- Conservative
- Extremely conservative

Do you have children?

- No
- Yes

Do you plan to have children/more children than you currently have?

- No
- Yes
- Don't know

Do you live with kids under 12 years of age, such as your own children or grandchildren, nephews, or siblings?

- No
- Yes

Do you have pets?

- No
- Yes

Have you or anyone in your family suffered from the consequences of lead poisoning?

- No
- Yes
- Don't know

Had you heard about lead service lines before this survey?

- No, I had never heard about lead service lines.
- Kind of, I had heard about lead in plumbing, but not about lead service lines.
- Yes, I had heard about lead service lines.

Powered by Qualtrics
